# Exploring parental knowledge, care-seeking, and support strategies for neonatal illness: an integrative review of the African Great Lakes region

**DOI:** 10.1080/16549716.2025.2450137

**Published:** 2025-02-03

**Authors:** Sarah Farrell, Tracey A. Mills, Dame Tina Lavender

**Affiliations:** Centre for Childbirth, Women’s, and Newborn Health, International Public Health, Liverpool School of Tropical Medicine, Liverpool, UK

**Keywords:** Neonatal danger signs, neonatal illness, midwifery, integrative review, sub-Saharan Africa, care-seeking

## Abstract

**Background:**

Sub-Saharan Africa shoulders much of the global burden of neonatal mortality. Quality postnatal care is often lacking due to availability, accessibility, mistrust of health systems, and socio-economic barriers, yet delays in care-seeking contribute to avoidable neonatal deaths. Research highlights the urgent need for improved health education about neonatal illness; however, contextual factors are rarely considered, and few interventions have been implemented.

**Objectives:**

To critically examine the literature on parents’ knowledge of neonatal illness and care-seeking behaviour and evaluate interventions supporting parental understanding in sub-Saharan African Great Lakes countries.

**Methods:**

Systematic searches were conducted in CINAHL, MEDLINE, Global Health, the Cochrane Library, and thesis repositories. Studies meeting inclusion criteria were critically analysed using Whittemore and Knafl’s framework, and quality was assessed with Hawker et al.’s tool, following PRISMA guidelines.

**Results:**

Seventy studies (48 quantitative, 14 qualitative, eight mixed methods) were reviewed. The first theme, “poor knowledge of neonatal illness”, showed parents struggled to recognise illness, with knowledge affected by maternity and socio-economic factors. The second theme, “sub-optimal healthcare-seeking behaviour”, highlighted delayed care-seeking due to cultural, social, and economic factors. Finally, “strategies to support parents’ understanding” emphasised the roles of community workers, health education phone calls, SMS, and videos, and neonatal monitoring systems.

**Conclusions:**

Parental knowledge of neonatal illness is generally low, and care-seeking is influenced by beliefs, trust in healthcare, and logistical challenges. While community health workers and multi-media interventions appear effective, health education efforts must address contextual barriers and beliefs to improve recognition and care-seeking for neonatal illness.

## Paper Context

- **Main findings**: Parents lack understanding of neonatal illness and often do not seek timely and appropriate care, yet few interventions have been implemented to support their understanding.
- **Added knowledge**: Care-seeking behaviour is complex; while it is related to knowledge of neonatal illness it is also affected by cultural and practical barriers.
- **Global health impact for policy and action**: Interventions to support parents to recognise neonatal illness must consider the cultural and familial context of knowledge acquisition and care-seeking.

## Background

High neonatal mortality rates across the African Great Lakes region reflect the significant challenges these countries face in enhancing neonatal health [1]. To meet the Sustainable Development Goal targets, it is imperative to implement measures that accelerate progress in reducing neonatal mortality [2]. The provision of quality care during facility-based births and immediate postnatal care is vital in mitigating the risk of neonatal mortality [3]. The first 24 hours post-birth are critical, accounting for nearly half of neonatal deaths, thus necessitating close monitoring to detect complications [4]. International guidelines recommend that newborns remain in health facilities for at least 24 hours [5]. However, many newborns are discharged within this period, often due to pressures within health facilities [6,7]. Despite an increase in the proportion of facility births across sub-Saharan Africa, a substantial number of women continue to give birth at home without support from qualified health professionals [8]. As over half of neonatal deaths occur after the first 24 hours of life, ongoing postnatal contact with qualified health professionals is advised. In addition to an early examination in the health facility or at home, three further postnatal contacts are recommended between 48 and 72 hours, between seven and 14 days, and during week six postpartum, before vaccinations commence [5]. This contact facilitates the prompt identification of complications and the communication of essential health advice [9]. Nevertheless, postnatal care has not received the same level of attention globally as antenatal and skilled birth care [10]. A recent multinational analysis revealed that less than a quarter of neonates in sub-Saharan Africa receive adequate postnatal care [11]. The low utilisation of postnatal care is often attributed to financial, cultural, and communication factors [9]. However, the bigger picture is complex, with systems unable to provide sufficient postnatal contacts with qualified health workers [12], and a lack of understanding among women regarding its importance [13].

Parents play a crucial role in identifying neonatal illness, particularly in the absence of regular postnatal care or between healthcare contacts. Many previous studies have assessed parents’ knowledge of specific symptoms known as neonatal danger signs (NDS). The NDS are defined within World Health Organisation postnatal guidance, which emphasises the importance of monitoring neonates for poor feeding, convulsions, fast breathing, severe chest in-drawing, no spontaneous movement, fever, hypothermia, jaundice in the first 24 hours, or any yellow palms and soles [5]. A growing body of evidence from the African Great Lakes countries, and other lower- and middle-income countries, indicates that parents often lack the necessary knowledge to identify NDS and respond appropriately. For example, a systematic review of studies conducted in Ethiopia revealed low maternal awareness of NDS and recommended enhancing antenatal and postnatal care attendance, alongside increased community-based health education [14]. Additionally, research on care-seeking behaviour related to NDS has highlighted significant delays. In a Kenyan study, while half of the mothers sought care within an hour of recognising their newborn’s illness, almost a third delayed for more than six hours [15]. In Uganda, researchers estimated that delays in recognising NDS and deciding to seek care contributed to half of the neonatal deaths [16]. Despite the evidence that parents often lack adequate understanding of neonatal illness and often demonstrate suboptimal care-seeking behaviour, there remains a gap in knowledge regarding the best methods, personnel, timing, or tools to support understanding and appropriate care-seeking.

## Aim

To identify and synthesise existing literature surrounding understanding of newborn illness or of ‘neonatal danger signs’ among parents of neonates in the Great Lakes countries of sub-Saharan Africa.

## Objectives

1. To explore parents’ understanding of neonatal illness and the barriers or facilitators to this understanding
2. To explore care-seeking behaviour for neonatal illness, including any barriers or facilitators
3. To explore evidence on strategies to support parents’ understanding of neonatal illness

## Methods

### Study design

An integrative review method was used to identify and synthesise research from different paradigms [17]. Whittemore and Knafl’s [18] rigorous framework was followed including (1) problem identification; (2) literature search; (3) data evaluation; (4) data analysis; and (5) presentation of the results. The PRISMA statement guided the reporting [19].

### Literature search

Comprehensive and systematic searches of the Cumulative Index to Nursing and Allied Health Literature (CINAHL), Medical Literature Analysis and Retrieval System Online (MEDLINE), Global Health, and Cochrane Library databases were conducted from 27^th^ November to 11 December 2023. These used keyword combinations (Table 1), common Boolean operators and database subject headings. Thesis repositories were searched using the neonatal keywords.

**Table 1.** Keyword combination used for searching.

Search ID#	Keywords
S1	“neonatal danger sign*’ OR “new?born danger sign*” OR “neonatal illness*” OR “new?born illness*” OR “new?born complication*” OR ’ neonatal complication*” OR ‘sick neonate’ OR ‘sick infant’
S2	parent* OR mother* OR father* OR women* OR men* OR caregiver OR ‘primary care?taker’ OR maternal OR partner* OR husband*
S3	knowledge OR understanding OR recogni* OR observ* OR concern* OR identif* OR assess* OR beliefs OR detection OR repon* OR experience OR involvement OR ‘decision?making’ OR ‘care?practices’ OR ‘care?seeking’ OR ‘health?seeking’ OR ‘treatment?seeking’ OR ‘treatment practices’ OR utili*ation OR ‘illness narrative’
S4	‘sub?Saharan Africa*’ OR Kenya OR Burundi OR Congo OR Ethiopia OR Malawi OR Mozambique OR Rwanda OR Zambia OR Tanzania OR Uganda
S5	S1 AND S2 AND S3 AND S4

All papers were screened by SF using the title, abstract, and where necessary full text, following the inclusion and exclusion criteria (Table 2). Studies from the African Great Lakes countries were eligible, including Kenya, Burundi, Congo, Ethiopia, Malawi, Mozambique, Rwanda, Zambia, Tanzania, and Uganda.

**Table 2.** Inclusion and exclusion criteria.

PICO/PICo	Inclusion Criteria	Exclusion Criteria
Population	Parents (mother, father, or both) who had a neonate within the previous two years	Parents with children older than two years
		Parents with babies in NICU
Intervention or Phenomenon of Interest	Understanding of neonatal illness or ‘neonatal danger signs’	Childhood illness
	Sources of knowledge about neonatal illness	
	Barriers and facilitators to understanding of neonatal illness	
	Care seeking behaviour related to neonatal illness	
	Barriers and facilitators to care seeking for neonatal illness	
	Interventions to support recognition of neonatal illness or care seeking for neonatal illness	
Comparison or Context	Studies with or without comparative groups	
	Studies in Great Lakes countries of sub-Saharan Africa; Burundi, the Democratic Republic of the Congo, Ethiopia, Kenya, Malawi, Mozambique, Rwanda, Tanzania, Uganda, and Zambia	
	Studies in health or community settings	
	Published since 2013	
	Written in English or Swahili	
Outcome	Understanding or knowledge about neonatal illness	
	Sources of information about neonatal illness	
	Health system related barriers and facilitators	
	Socio-economic barriers or facilitators	
	Existing interventions	

**Figure 1.**
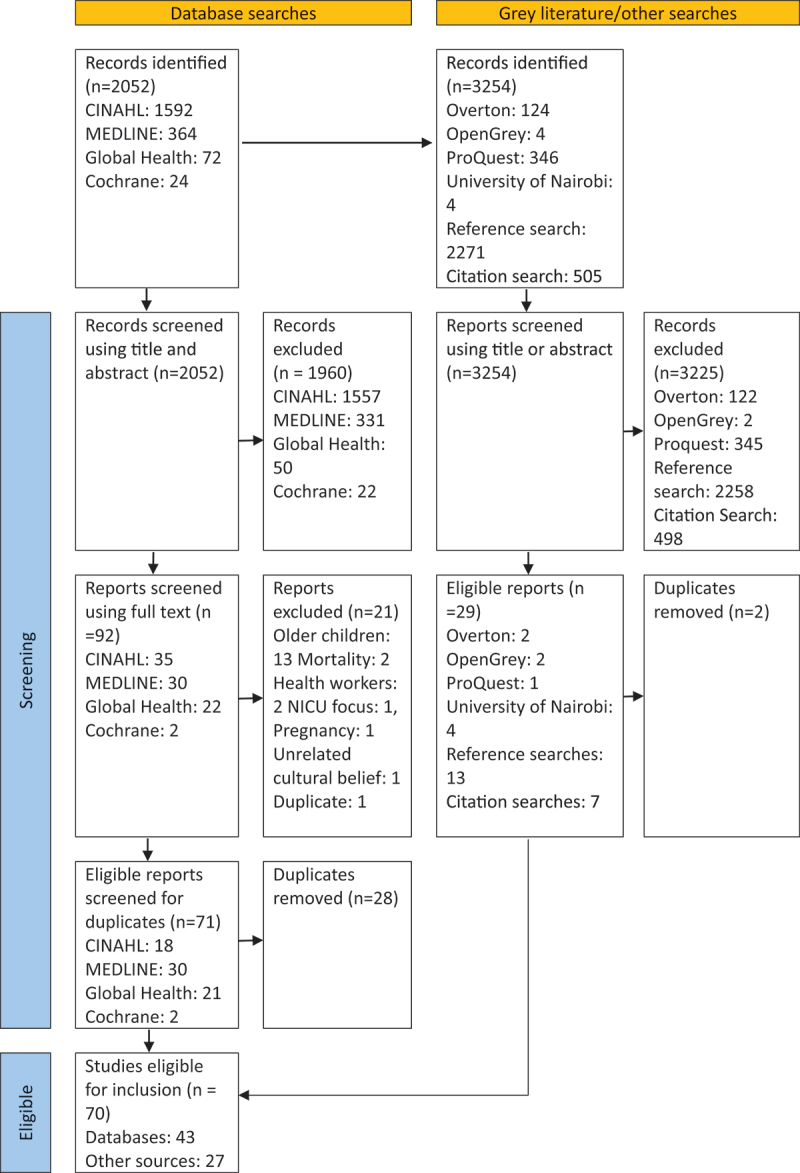
PRISMA flowchart process for selecting papers.

Thesis repositories, reference and citation searching between 11 December 2023 and 15 June 2024 identified further papers using the same eligibility criteria. The identification of eligible studies is summarised in Figure 1.

### Data evaluation

Hawker et al.’s [20] appraisal tool was used to assess the quality of papers across different paradigms. This assessed the abstract/title, introduction/aims, method, sampling, data analysis, ethics/bias, results, transferability/generalisability, and implications/usefulness. Each component was scored from good (4), to very poor (1) by SF. Scores above 30 indicated high quality, 24–29 were medium quality, and below 24 were low quality [21].

### Data analysis

Initially, papers were grouped as being related to parents’ knowledge, care-seeking, or including interventions. Studies were thoroughly reviewed, relevant characteristics extracted to tables, and recurring themes analysed. As data were organised, themes were refined iteratively, and findings underwent rigorous critical analysis.

## Presentation of findings

Seventy papers met the inclusion criteria, these included studies conducted in Ethiopia (47), Kenya (7), Uganda (6), Tanzania (3), Zambia (3), Rwanda (2), Democratic Republic of Congo (1), and multi-country (1). There were 48 quantitative studies (Table 3), 14 qualitative studies (Table 4), and eight mixed-methods studies (Table 5).

**Table 3.** Quantitative studies included.

Author, date, country	Title	Study design + method	Aim	Sample	Main findings	Quality score/36
Adem et al., 2017, Ethiopia	Awareness and Associated Factors Towards Neonatal Danger Signs Among Mothers Attending Public Health Institutions of Mekelle City, Tigray, Ethiopia, 2015	Cross-sectional study Structured questionnaire	Assess mothers’ awareness of and factors associated with NDS	350 mothers of babies aged up to 28 days attending health institutions in Mekelle City	50% had good knowledge (3+ NDS) Birth preparedness + postnatal care was associated with knowledge of NDS 74% sought medical care for identified NDS Birth at facility and good essential newborn care practices associated with good care-seeking behaviour Reasons for not seeking medical care were lack of knowledge of options, preferring home treatment, money, distance, belief no treatment available for neonates	33
Alkizim et al., 2018, Kenya	Efficacy of mobile phone use on patient retention in care in postnatal clinic in Nakuru	Randomised Controlled Trial Random allocation to receive text messages, phone calls, or usual care	Assess use of mobile phones as an intervention to increase postnatal clinic retention, promote exclusive breastfeeding, and increase mothers’ knowledge of NDS	180 postnatal mothers with infants from Nakuru County Hospital	Receiving text or phone reminders significantly increased knowledge of NDS and postnatal care attendance Mean score of NDS knowledge in was 43% in the control arm, 76% text group, and 80% phone group	34
Amolo, undated, Kenya	Knowledge and attitude of postnatal mothers on essential newborn care practices at Kenyatta National Hospital	Cross-sectional study Questionnaire with open and closed ended questions	Assess postnatal mothers’ knowledge of essential newborn care including NDS	380 mothers on postnatal ward in Nairobi	Almost all mothers recognised fever as a NDS Few women received NDS education during pregnancy or at delivery Antenatal care visits and education about NDS were associated with increased knowledge of NDS	31
Anmut et al., 2017, Ethiopia	Mother’s knowledge and Practice about Neonatal Danger Signs and Associated Factors in Wolkite Town, Gurage Zone, SNNPR, Ethiopia, 2017	Cross-sectional study Structured questionnaire	Assess mothers’ knowledge and practice about NDS	355 mothers who had given birth in previous 12 months living in Wolkite Town	79% had heard of NDS Higher educational level, higher income, facility birth, and health worker as source of NDS information were associated with good NDS knowledge 65% had unsafe practices for a sick neonate Husbands’ educational level, husbands’ occupation, facility birth, and postnatal care attendance were associated with good care-seeking	32
Asnakew et al., 2018, Ethiopia	Level of Knowledge About Neonatal Danger Signs and Associated Factors Among Mothers Who Delivered at Home in Fogera District, South West, Ethiopia	Cross-sectional study Structured questionnaire	Assess mothers’ knowledge of NDS	845 mothers with babies under six months living in selected villages of Fogera District	64% had good knowledge (3+ NDS) Partner involvement in antenatal care and media access were associated with good knowledge	33
Bekele et al., 2020, Ethiopia	Mothers’ knowledge and their health seeking behaviour about neonatal danger signs and associated factors in Fiche town, Oromia region, Ethiopia	Cross-sectional study Structured questionnaire	Assess mothers’ risk factors associated with knowledge and health-seeking behaviour about NDS	360 mothers living in Fiche Town with a baby under 12 months	52% had good knowledge (3+ NDS) Antenatal care attendance + higher education associated with good NDS knowledge 53% sought healthcare for NDS symptoms Barriers to care-seeking were cost, lack of understanding, being busy	34
Berhane et al., 2018, Ethiopia	Parents’ Knowledge of Danger Signs and Health Seeking Behavior in Newborn and Young Infant Illness in Tiro Afeta District, Southwest Ethiopia: A Community-based Study	Cross-sectional study Structured questionnaire	Assess mothers’ knowledge and health seeking behaviour for NDS	422 mothers with babies under 6 months living in Tiro Afeta District	Few women received NDS education during antenatal or postnatal care Occupation and higher income associated with good knowledge of NDS 93% intended to use a health centre if baby got sick Care-seeking more likely if baby born in a facility and when parents able to make decisions	32
Bogale et al., 2018A, Ethiopia	Mothers’ treatment seeking intention for neonatal danger signs in northwest Ethiopia: A structural equation modelling	Cross-sectional study Structured interviews	Test a theoretical model of factors affecting mothers’ treatment seeking intention for NDS	2158 pregnant women and women who had a baby under 6 months, or had a stillbirth in northwest Ethiopia	Antenatal care attendance correlated with knowledge of NDS Knowledge of NDS, positive perceived behaviour of health care providers, household level woman empowerment, and perceived cost of treatment showed direct, positive and significant association with treatment-seeking intention Distance and socio-economic status were not related to treatment-seeking intention	34
Borde et al., 2020, Ethiopia	Incidence of postpartum and neonatal illnesses and utilization of healthcare services in rural communities in southern Ethiopia: A prospective cohort study	Prospective cohort study Home visits to assess illness episodes and care-seeking behaviour	Assess the incidence and risk factors for postpartum and neonatal illnesses and measure the utilization of healthcare services	784 postpartum women and 772 neonates in selected rural kebeles of southern Ethiopia	Very low care-seeking behaviour Belief illness was not serious, would resolve on its own, didn’t think the neonate had an illness, and cost hindered care-seeking	34
Bulto et al., 2019, Ethiopia	Knowledge of neonatal danger signs, care seeking practice and associated factors among postpartum mothers at public health facilities in Ambo town, Central Ethiopia	Cross-sectional study Face to face interviews	Assess knowledge of NDS, care-seeking practice and associated factors among postpartum mothers	404 postpartum mothers attending first immunisation appointment in Ambo Town	20% of mothers had good knowledge (3+ NDS) Higher education, recent experience of NDS, attending postnatal care, and receiving NDS education were associated with knowledge 60% sought care immediately (of the 10% that had a sick baby)	34
Buser et al., 2021, Zambia	Maternal knowledge of essential newborn care in rural Zambia	Two-group comparison design Structured questionnaire	Compare maternal knowledge of newborn care for women referred from facilities with and without maternity waiting homes	250 mothers including users of maternity waiting homes and those that did not use them in rural Zambia	Mother most likely to seek care if baby has fever No significant differences between women who attended maternity waiting homes and those who did not	33
Chanie, 2019, Ethiopia	Knowledge and Health Care Seeking Behaviour About Neonatal Danger Signs Among Mothers Visiting Immunization Unit in Public Health Facilities of Debre Markos Town Northwest Ethiopia, June 2016	Cross-sectional study Structured questionnaire	Assess knowledge and health care seeking behaviour about NDS among mothers	285 mothers with infants under 1 year attending public immunization clinics in Debre Markos	Less than half mothers were highly knowledgeable about NDS 81% sought health care for NDS of which 47% was within 4 hours Delayed care seeking due to belief in spontaneous resolution, lack of recognition, cost, alternative therapies, and distance Parents lacked understanding of causes of NDS	34
Degefa et al., 2019, Ethiopia	Knowledge about Neonatal Danger Signs and Associated Factors among Mothers Attending Immunization Clinic at Arba Minch General Hospital, Southern Ethiopia: A Cross-Sectional Study	Cross-sectional study Structured questionnaire	Assess knowledge about neonatal danger signs and associated factors among mothers	364 mothers with infants under 12 months attending Arba Minch Hospital immunization clinic	40% had good knowledge (2+ NDS) Fever was most known (33%) but all scores low Higher educational level and postnatal care attendance associated with good NDS knowledge	34
Dida et al., 2024, Ethiopia	Awareness and healthcare seeking behavior of neonatal danger signs, and predictor variables among mothers/caregivers in four developing regional state of Ethiopia	Cross-sectional study Structured questionnaire	Assess mothers’ awareness and health care seeking behaviour for NDS	6706 mothers/caregivers of children below five years of age from the four developing regional states of Ethiopia	Refugees less likely to have good knowledge of NDS Muslims more likely than Christians, and mothers empowered to attend health facilities without permission, were more likely to have good knowledge of NDS Antenatal care attendance and institutional birth were associated with good care-seeking behaviour	29
Gage et al., 2022, DRC	Impact of the Momentum pilot project on male involvement in maternal health and newborn care in Kinshasa, Democratic Republic of the Congo: a quasi-experimental study	Quasi-experimental community-based pilot study design 3 intervention and 3 comparison health zones	Assess the impact of the Momentum project on male involvement in maternal health and newborn care	1204 male partners of first-time mothers with live births living in intervention or control areas in Kinshasa	Intervention, which included family planning, maternal and newborn health and nutrition elements was associated with 13.9% point increase in knowledge of NDS, compared with control zones Home visits, group education and social/behavioural change communication were effective methods to increase NDS knowledge	35
Gebremeskel et al., 2021, Ethiopia	Effect of place of birth on knowledge of neonatal danger signs and associated factors among mother’s in Meicha district, Northwest Ethiopia: A community-based comparative cross-sectional study	Cross-sectional study Structured questionnaire	Compared knowledge of NDs between mothers who delivered at home and in health facilities	650 mothers who had given birth in previous 2 months living in Meicha districts	60% of mothers who delivered in health facility had good knowledge of NDS compared with 41% of mothers who gave birth at home Living close to facilities, increased parity, and birth in health facility were all associated with good NDS knowledge	31
Gebretsadik et al., 2018, Ethiopia	Home-based neonatal care by Health Extension Worker in rural Sidama Zone southern Ethiopia: a cross-sectional study	Cross-sectional study Structured questionnaire	Evaluate the prevalence and timing of home-based neonatal care by community health workers	2040 mothers who had given birth in previous 6 months living in Sidama Zone	12% of mothers saw community health workers after birth but only 32% of these received NDS education Health care most likely to be sought for fever, poor feeding or fast breathing	32
Getachew et al., 2022, Ethiopia	Determinants of maternal knowledge on neonatal danger signs and care-seeking practices in a rural area of southeastern Ethiopia	Cross-sectional study Structured questionnaire	Assess the determinants of maternal knowledge of neonatal danger signs and care-seeking practices	520 postnatal mothers who had given birth within previous 6 months and resident for at least 6 months in Chole Woreda	50% had good knowledge (3+ NDS) NDS was the least included topic of antenatal education Higher educational level, urban residency, attending antenatal care, receiving antenatal education, and essential newborn care knowledge were associated with knowledge of NDS 61% sought care in health facility 47% preferred traditional healer	34
Guta et al., 2020, Ethiopia	Knowledge of Neonatal Danger Signs and Associated Factors Among Mothers of <6 Months Old Child in Dire Dawa, Ethiopia: A Community Based Cross-Sectional Study	Cross-sectional study Face to face interview	Assess mothers’ knowledge of neonatal danger signs and associated factors	699 mothers with a baby under 6 months of age residing in the selected kebeles in Dire Dawa	41% had good knowledge (3+ NDS) Health workers were most likely source of knowledge Government employees, attendance at antenatal care, fathers with higher education, experience of NDS, and having NDS counselling were associated with good knowledge Of those with experience of NDS, 62% sought care immediately	34
Hunde et al., 2023, Ethiopia	What mothers know about neonatal danger signs: A Cross Sectional Study of Ethiopia	Cross-sectional study Semi-structured questionnaire	Assess mothers’ knowledge and associated factors	727 mothers with babies under 12 months living in Nekemte	56% knew 5+ NDS Postnatal counselling rarely included NDS Married status, higher educational level, antenatal care attendance, postnatal care attendance, facility birth, TV and internet access were associated with knowledge of NDS	34
Idris et al., 2022, Ethiopia	Healthcare-Seeking Behaviour and Associated Factors for Newborn Danger Signs among Mothers Who Gave Birth in the Last 12 Months in Anlemo District	Cross-sectional study Structured questionnaire	Assess mothers’ healthcare seeking behaviour for NDS and associated factors	442 mothers with babies under 12 months living in selected kebeles of Anlemo District	63% had adequate knowledge (3+ NDS) Community health workers were most likely source of knowledge Of mothers with experience of NDS, 35% had sought healthcare Majority of mothers sought non-medical care More likely to seek care if higher education level, lived near facility, or gave birth in facility Barriers cited were belief in alternative treatment, distance, poor view of health workers, not thinking condition serious, busyness	34
Jemberia et al., 2018, Ethiopia	Low level of knowledge about neonatal danger signs and its associated factors among postnatal mothers attending at Woldia general hospital, Ethiopia	Cross-sectional study Interviewer administered questionnaire	Assess mothers’ knowledge about neonatal danger signs and its associated factors	197 mothers attending postnatal care at Woldia General Hospital	88% of mothers identified < 6 NDS 27% received NDS education during postnatal care Age, higher education, antenatal care attendance, urban residence, and having NDS counselling were associated with knowledge	33
Kananura et al., 2017, Uganda	Effect of a participatory multisectoral maternal and newborn intervention on birth preparedness and knowledge of maternal and newborn danger signs among women in Eastern Uganda: a quasi-experiment study	Quasi-experimental pre-post comparison design Structured questionnaires	Assess the effect of a participatory multi-sectoral maternal and newborn intervention on birth preparedness and knowledge of obstetric danger signs among women in Eastern Uganda	Mothers who delivered in previous 12 months living in study area − 2237 at baseline (1101 comparison, 1136 intervention groups). 1946 at endline (920 comparison, 1026 intervention groups)	Intervention including community health worker home visits, radio messages, savings groups, and linking local transport with savings groups increased knowledge of NDS Knowledge increased from 37% to 65% in the control area and from 43% to 91% in the intervention area; a significant intervention contribution of 20%	35
Kassaw et al., 2021, Ethiopia	Evidence from 2016 Ethiopian demographic and health survey data: association between post health education maternal knowledge and neonatal danger signs	Secondary data analysis Data extraction from Ethiopian DHS 2016 of questions addressing post health education maternal knowledge of NDS	Assess maternal knowledge about neonatal danger signs and its associations	325 taken from 2016 EDHS data	70% had low knowledge (score less than median) Factors associated with good knowledge were not wanting more children and higher education	27
Kebede et al., 2022, Ethiopia	Mothers experience on neonatal danger signs and associated factors in northwest Ethiopia: a community based cross-sectional study	Cross-sectional study Semi-structured questionnaire	Estimate mothers’ experience of NDS and its associated factors in Northwest Ethiopia	2424 women who had given birth in past 6 months from the chosen clusters	Only 0.6% knew 3+ NDS Urban living, higher education, increased distance, and not having immunization were associated with higher knowledge of NDS Attending antenatal or postnatal care were not associated Few had experienced NDS but 66% with experience went to health centre first	31
Kebede et al., 2021, Ethiopia	Married women’s decision-making autonomy in the household and maternal and neonatal healthcare utilization and associated factors in Debretabor, northwest Ethiopia	Cross-sectional study Structured questionnaire	Assess married women’s decision-making autonomy including on maternal and neonatal healthcare utilization and associated factors	730 married women with an infant under 1 year residing in the selected kebele in Debretabor	84% decided as a couple to take sick newborn to health facility Older mothers, higher income, husband involvement in maternal and neonatal care, and adequate NDS knowledge all associated with higher decision-making ability	35
Kibaru + Otara, 2016, Kenya	Knowledge of neonatal danger signs among mothers attending well baby clinic in Nakuru Central District, Kenya: cross sectional descriptive study	Cross-sectional study Structured questionnaire	Determine mothers’ level of knowledge of NDS and determine the associated factors	414 Mothers attending well baby clinics in Nakuru District with babies aged 6–9 weeks	16% identified 3+ NDS Fever most commonly known NDS No significant factors associated with knowledge on multivariate logistic regression	31
Masoi + Kibusi, 2019, Tanzania	Improving pregnant women’s knowledge on danger signs and birth preparedness practices using an interactive mobile messaging alert system in Dodoma region, Tanzania: a controlled quasi experimental study	Controlled quasi-experimental study Compared interactive mobile messaging service providing health education messages with usual care	Compare impact of intervention of SMS messages with control group on knowledge of obstetric and neonatal danger signs in Dodoma region	450 women recruited during pregnancy (150 intervention, 300 control) attending antenatal care at included facilities in Dodoma region	At baseline, 46% in the intervention group and 45% in the control group were knowledgeable about NDS After the intervention, there was a significant difference between groups with 77% in the intervention group and 48% in the control group knowledgeable about NDS They identified a Cohen’s d effect size of 85%	35
Masoi et al., 2020, Tanzania	The Pattern and Level of Knowledge on Obstetric and Newborn Danger Signs and Birth Preparedness among Pregnant Women in Dodoma Municipal: A Cross-Sectional Study	Cross-sectional study Semi-structured questionnaire	Understand the pattern and level of knowledge on obstetric and neonatal danger signs, birth/complication readiness among pregnant women	450 pregnant women attending antenatal care in health facilities in the Dodoma Municipal	45% had adequate knowledge of obstetric and neonatal danger signs (8+/25) Older age and higher education associated with good knowledge	29
Mersha et al., 2017, Ethiopia	Mother’s level of knowledge on neonatal danger signs and its predictors in Chencha District, Southern Ethiopia	Cross-sectional study Structured questionnaire	Assess level of knowledge about NDS among mothers	630 mothers with babies under 6 months of age in Chencha District	50% had good knowledge (3+ NDS) 52% aware of NDS from health workers Urban residence, radio ownership and good essential newborn care knowledge associated with good knowledge of NDS	33
Mesele et al., 2023, Ethiopia	Mothers’ health care seeking behaviour for neonatal danger sign in southern Ethiopia: Community based cross – sectional study	Cross-sectional study Structured questionnaire	Assess health care-seeking behaviour of mothers related to NDS	410 mothers who had given birth in the previous 12 months from Sodo town, Wolaita Zone	Of mothers with NDS experience, 48% used a health facility Husband’s education level, radio access, urban residence, antenatal attendance, and postnatal care attendance were associated with good knowledge of NDS Reasons for not seeking care were cost, belief not serious, belief in home remedies, poor view of health workers/facility, lack of knowledge of NDS	34
Molla et al., 2020, Ethiopia	Knowledge of neonatal danger signs among recently delivered mothers in Mekedella woreda, Northeast Ethiopia, in 2017: a cross-sectional study	Cross-sectional study Structured questionnaire	Assess mothers’ level of knowledge of NDS and associated factors	757 mothers with a baby under 12 months living within selected kebeles of Mekedella woreda	28% had good knowledge (3+ NDS) Health development army were most likely source of knowledge Higher education, urban residence, antenatal visits, facility birth, postnatal care attendance and education about NDS were all associated with knowledge of NDS	33
Mose et al., 2021, Ethiopia	Determinants of maternal knowledge of neonatal danger signs among postpartum mothers in Southern Ethiopia: institutional- based cross-sectional study	Cross-sectional study Structured questionnaire plus observational checklists	Assess knowledge of NDS and associated factors among postpartum mothers	608 postpartum mothers attending public health facilities in Southern Ethiopia	48% had good knowledge (3+ NDS) Jaundice was most known NDS Urban residence, antenatal care attendance, breastfeeding education, postnatal education, increased parity, and good essential newborn care practice were associated with good knowledge of NDS	35
Mujawimana et al., 2020, Rwanda	Parents’ Knowledge of Neonatal Danger Signs and Associated Factors at Health Centers in Kigali, Rwanda	Cross-sectional study Self-administered structured questionnaire	Assess mothers’ knowledge of NDS	209 parents of neonates (up to 28 days) attending health centres in Kigali	Health workers were the most frequent source of information about NDS Higher educational level, increased parity, public hospital use, health worker source of information was associated with good NDS knowledge	29
Nigatu, Worku + Dadi, 2015, Ethiopia	Level of mother’s knowledge about neonatal danger signs and associated factors in North West of Ethiopia: a community based study	Cross-sectional study Structured questionnaire	Determine the level of mothers’ knowledge about neonatal danger signs and to identify factors associated with good knowledge	603 mothers who had given birth in previous 6 months in study areas of NW Ethiopia	18% had good knowledge (3+ NDS) Higher educational level, attending antenatal care, attending postnatal care or having TV access were associated with good knowledge of NDS	34
Njuguna et al., 2018, Kenya	Effectiveness and acceptability of audiovisual aids for increasing knowledge of neonatal danger signs among primiparous women: A randomised controlled trial	Randomised Controlled Trial One postnatal ward had intervention, and one postnatal ward was the control with follow up by phone on day 7 and day 28	Assess effectiveness and acceptability of audiovisual aids for increasing knowledge of NDS among primiparous women in Nairobi	153 mothers on postnatal wards at Kenyatta Hospital	Mothers found postnatal videos beneficial and easy to follow Knowledge of some specific NDS was higher in the intervention group (videos) after one and four weeks compared to control (usual care) Knowledge of NDS reduced in both groups between one and four weeks	31
Roney et al., 2021, Kenya	Men’s and women’s knowledge of danger signs relevant to postnatal and neonatal care seeking: A cross sectional study from Bungoma County, Kenya	Cross-sectional study Structured questionnaire	Assess knowledge of NDS relevant to care seeking.	Women who had recently given birth (*n* = 348) and men whose wives had recently given birth (*n* = 82) living in Bungoma County	51% women, 50% men knew 1+ NDS Women more likely to identify specific NDS than men, but followed same pattern Women’s knowledge associated with higher education, higher income, multigravidity, older age at first pregnancy Men’s knowledge associated with higher income only Of those who experienced NDS 53% sought care quickly	33
Sandberg et al., 2014, Uganda	Inadequate knowledge of neonatal danger signs among recently delivered women in southwestern rural Uganda: a community survey	Community survey/cross-sectional study Community survey (pre-intervention study) using questionnaire	Explore the knowledge of key newborn danger signs among mothers	765 recently delivered women in southwestern Uganda	15% knew 2+ NDS Mothers thought NDS included false teeth and millet disease Area of residence, age, marital status, educational level, household assets, parity, distance to health facility, antenatal are attendance, skilled birth attendant for delivery were NOT associated with good knowledge of NDS	32
Shitu et al., 2021, Ethiopia	Knowledge of neonatal danger signs and associated factors among husbands of mothers who gave birth in the last 6 months in Gurage Zone, Southern Ethiopia, 2020: a community- based cross- sectional study	Cross-sectional study Structured questionnaire	Assess NDS knowledge of fathers in Gurage Zone	633 husbands of mothers who gave birth within 6 months living in Gurage Zone	40% of husbands had good knowledge (knew 5+ NDS) Factors associated with good knowledge were urban residence, attending antenatal care with wife, having more children, having education about NDS from health worker, higher educational level	33
Tekulu, 2017, Ethiopia	Knowledge of neonatal danger signs and associated factors among mothers how gave birth in the last four months attending immunization services in Harar Town public health facilities, Ethiopia	Cross-sectional study Structured questionnaire	Assess mothers’ knowledge of NDS and associated factors	432 mothers attending immunization services in Harar Town who gave birth within last 4 months	33% knew 3+ NDS 91% attended antenatal care but only 34% had NDS counselling Higher education level, postnatal care, caesarean birth, counselling about NDS during antenatal care, and multiparity were associated with knowledge of NDS	34
Tesfau et al., 2022, Ethiopia	Effect of health facility linkage with community using postnatal card on postnatal home visit coverage and newborn care practices in rural Ethiopia: a controlled quasi-experimental study design	Controlled quasi-experimental study Women received interactive mobile messaging service or usual care	Compare impact of intervention of SMS messages with control group receiving usual care on knowledge of obstetric and neonatal danger signs	705 baseline data and 980 endline data sets of mothers who delivered in the previous 12 months	Use of postnatal cards by Health Extension Workers and facility capacity strengthening was effective to increase knowledge of NDS Good knowledge of NDS (3+) increased by 13.6%	35
Tesfaye et al., 2022, Ethiopia	Maternal Health Care Seeking Behaviour for Neonatal Danger Signs and Associated Factors Among Post-Partum Mothers in Southeast Ethiopia: A Cross-Sectional Study	Cross-sectional study Structured questionnaire	Assess maternal health-seeking behaviour for NDS and associated factors	400 women whose baby had been ill in first 28 days of life	51% had good knowledge (4+ NDS) Higher educational level associated with good knowledge of NDS 44% sought care for NDS within 1 day 76% of mothers had decision-making power Postnatal care, knowledge of NDS, decision-making power, having a partner, or having health insurance were associated with good care-seeking behaviour Reasons for delayed care seeking were belief it would resolve, lack of belief in medicine, belief in traditional treatment, cost, lack of decision making, workload, transport, distance, long waiting times in facilities	34
Welay et al., 2019, Ethiopia	Knowledge of neonatal danger signs and associated factors among mothers who gave birth during the last 4 months while attending immunization services in Harar town public health facilities, Ethiopia, 2017	Cross-sectional study Structured questionnaire	Assess mothers’ knowledge of NDS and associated factors	432 mothers attending immunization services in Harar Town who gave birth within last 4 months	33% knew 3+ NDS Education about NDS was the lowest of all education areas NDS information received from health workers, media, family, books, friends Higher education, increased parity, early initiation of breastfeeding, or received education about NDS were associated with good NDS knowledge	31
Wilmot et al., 2017, Rwanda	Missed opportunities in Neonatal Deaths in Rwanda: Applying the Three Delays Model in a Cross-Sectional Analysis of Neonatal Death	Cross-sectional study Standardised audit questionnaire examined neonatal deaths recorded in the MOH Neonatal Deaths audit database	Use the three-delay model to understand why neonates die in Rwanda	1324 neonatal deaths which occurred in a health facility	Delays in care-seeking were associated with over 40% of deaths Only 26% neonates did not experience delays	34
Wudu et al., 2024, Ethiopia	Level of knowledge about neonatal danger signs and associated factors among postpartum mothers in public hospitals, northeastern Ethiopia	Cross-sectional study Semi-structured questionnaire, observation, chart reviews	Assess level of NDS knowledge among mothers	421 postpartum mothers in public hospitals in Amhara region	37% knew 3+ NDS 50% of those who had heard of NDS had been informed by health worker Higher education level, occupation outside home, breastfeeding education, and increased family size were associated with knowledge of NDS	33
Yadeta et al., 2018, Ethiopia	Antenatal care utilization increases the odds of women knowledge on neonatal danger sign: a community-based study, eastern Ethiopia	Cross-sectional study Structured questionnaire	Determine womens’ knowledge on key NDS and associated factors among	757 mothers who had given birth within 2 years from selected kebeles in Eastern Ethiopia	9% knew 4+ NDS Fever most commonly known NDS but knowledge of all NDS was low Attending antenatal care was associated with good knowledge of NDS	32
Yitayew et al., 2021, Ethiopia	Knowledge of neonatal danger signs and associated factors among mothers attending pediatric immunization clinics in Gidan District Health Centers, North Wollo, Ethiopia	Cross-sectional study Structured questionnaire	Assess the knowledge of neonatal danger signs and associated factors among mothers	399 mothers attending immunization clinics in health centres in Gidan District	48% had good knowledge (3+ NDS) 62% received NDS education Educational level, postnatal care attendance, NDS counselling, and experience of NDS were associated with good knowledge of NDS	34
Yosef et al., 2020, Ethiopia	Knowledge of Neonatal Danger Signs and Its Associated Factors among Mothers Attending Child Vaccination Centers at Sheko District in Southwest Ethiopia	Cross-sectional study Structured questionnaire	Assess the knowledge of neonatal danger signs and its associated factors among mothers	351 mothers who gave birth in the last year attending vaccination clinics in Sheko District	39% had good knowledge (3+ NDS) 72% knew fever Mothers older age, higher educational level, antenatal care attendance, and postnatal care attendance were associated with good knowledge of NDS	34

**Table 4.** Qualitative studies included.

Author, date, country	Title	Study design + method	Aim	Sample	Main findings	Quality score/36
Amare, Paul + Sibley, 2018, Ethiopia	Illness recognition and appropriate care seeking for newborn complications in rural Oromia and Amhara regional states of Ethiopia	Qualitative Illness narrative interviews with caregivers and witnesses to a neonatal illness event where some of the neonates died and some survived	Assess recognition of and timely biomedical care seeking for maternal + newborn complications	16 primary caregivers and ‘witnesses’ to cases where the newborn was alive at 28 days of life and 13 cases where the newborn had died within 28 days from Oromia and Amhara regions	NDS symptoms often experienced/mentioned in clusters Traditional beliefs about illness prevalent Folk belief ‘fallen uvula’ was common Perceived severity of illness not related to care-seeking Delayed care-seeking was common and related to beliefs about newborns, confinement period, hopelessness, hoping would resolve, poor communication and location of facilities	35
Bogale et al., 2018B, Ethiopia	Causal beliefs affect treatment practices and preferences for neonatal danger signs in Northwest Ethiopia: a qualitative study.	Qualitative Phenomenological qualitative study using in-depth interviews and focus group discussions	Explore beliefs and experiences of community members about the causes, treatment practices, and preferences for NDS	12 focus group discussions included 98 mothers, fathers and religious leaders. In-depth interviews included 6 community health workers and 30 pregnant women/women with babies under 6 months in northwest Ethiopia	Causes of neonatal illness attributed to local illnesses and beliefs Health Extension Workers believed some of the local beliefs Belief that non-medical care was superior Religious and cultural barriers to care-seeking, but often related to lack of understanding of NDS	34
Charlet et al., 2017, 7 countries	Summary findings from a mixed methods study on identifying and responding to maternal and newborn illness in seven countries: implications for programs.	Qualitative component of larger mixed-methods study Narrative interviews and focus group discussions	Illuminate the dynamics driving Delays 1 and 2 across seven countries for maternal and newborn illness and death	16 to 51 event narratives per country plus 0–20 key informant interviews or focus group discussions per country. Total 84 maternal illness cases, 45 maternal deaths, 83 newborn illness cases, 55 newborn deaths, 64 interviews/focus group discussions, and 99 health facility assessments	Prior experience helped parents recognise NDS Delay between symptom recognition and care-seeking was common Presence of NDS not linked to need for urgent action Fatalistic beliefs prevalent Sequential care seeking common	33
Nalwadda et al., 2015, Uganda	‘As soon as the umbilical cord gets off, the child ceases to be called a newborn’: sociocultural beliefs and newborn referral in rural Uganda	Qualitative Focus group discussions and interviews	Understand community perspective of barriers and facilitators to newborn referral	12 focus group discussions with parents, 11 interviews with Traditional Birth Attendants and mothers in rural Uganda	Routine confinement period while cord attached Care-seeking affected by support from local health workers, belief about illness, cost, previous negative experiences of facilities, weather Sequential care seeking common	33
Okuga et al., 2017, Uganda	Illness recognition and care-seeking for maternal and newborn complications in rural eastern Uganda	Qualitative Event narratives and focus group discussions	Explore illness recognition, decision making and appropriate care seeking for maternal/newborn illness	48 event narratives, 6 focus group discussions	Care seeking was complicated, often with many steps taken before hospital Newborn illness was often associated with non-medical causes Savings groups and community health worker access increased care-seeking Health worker rudeness and waiting times decreased care-seeking	34
Onarheim et al., 2017, Ethiopia	What if the baby doesn’t survive? Health-care decision making for ill newborns in Ethiopia	Qualitative In-depth interviews and focus group discussions	Examine families’ decision making and care seeking	41 interviews, 7 focus groups with primary caregivers who had experienced recent newborn illness or death, health workers, and community members in Butajira region	Care seeking was often delayed as a common approach was to wait and see Pros and cons of care-seeking weighed up and often affected by money, but also by low value of neonate as not considered a person Mothers most likely to want to seek care, but may not have sole decision making	34
Onarheim et al., 2020, Ethiopia	‘I wanted to go, but they said wait’: Mothers’ bargaining power and strategies in care seeking for ill newborns in Ethiopia	Qualitative In-depth interviews and focus group discussions	Explore mother’s roles in decision making for newborn illness	41 interviews, 7 focus groups with primary caregivers who had experienced recent newborn illness or death, health workers and community members in Butajira region	Mothers identified illness, but were not always able to make decisions Decisions often made based on cost Local practice of postnatal confinement reduced care seeking	34
Shamba et al., 2019, Tanzania	Delayed illness recognition and multiple referrals: a qualitative study exploring care-seeking trajectories contributing to maternal and newborn illnesses and death in southern Tanzania	Qualitative In-depth interviews and focus group discussions	Explore illness recognition, decision-making and care-seeking for cases of maternal and neonatal illness and death	48 interview participants, 5 focus groups with community leaders in Mtwara region	Participants found newborn illness difficult to recognise Severity of illness often misjudged Care-seeking often had may steps before reaching appropriate facility Barriers for care-seeking included husband being away, transport, lack of professionalism in facilities Traditional beliefs about causes of illness did not prevent care-seeking	35
Sivalogan et al., 2023, Zambia	Impact of beliefs on perception of newborn illness, caregiver behaviors, and care-seeking practices in Zambia’s Southern province	Qualitative In-depth interviews and focus group discussions	Assess how community beliefs influence newborn care behaviours, perception of illness and care seeking	339 women in 36 focus groups plus 42 in-depth interviews with key informants in Southern province	Advice sought from grandmother or traditional birth attendant for NDS, but parents usually made care-seeking decisions More likely to seek health care for malaria symptoms, abdominal pain, or cord infection Prevalent belief health facilities can not treat some conditions Cited barriers were distance, transport, cost, laziness, religious beliefs	35
Tareke et al., 2020, Ethiopia	Community’s perception, experiences and health seeking behavior towards newborn illnesses in Debre Libanos District, North Shoa, Oromia, Ethiopia: Qualitative study	Qualitative In-depth interviews and focus group discussions	Explore community members perception, experiences and health-seeking behaviour towards newborn illnesses	5 in-depth interviews with recent mothers, 7 key informant interviews, 3 focus group discussions in Debre Libanos District	Unspecific symptoms viewed as local illnesses which were treated with traditional medicine or home remedies Appropriate care seeking often delayed, but sometimes sought when baby deteriorated Care-seeking primarily for fever or breastfeeding problems	35
Tareke, Lemu + Feyissa, 2020, Ethiopia	Exploration of facilitators of and barriers to the community-based service utilization for newborn possible serious bacterial infection management in Debre Libanos District, Ethiopia: descriptive qualitative study	Qualitative In-depth interviews and focus group discussions	Explore barriers and facilitators to community-based service utilization for newborn infection	5 in-depth interviews with recent mothers, 7 key informant interviews, 4 focus groups with women and family members in Debre Libanos District	Dominant belief in traditional medicine so usually tried first Care-seeking affected by lack of understanding about treatment options for neonates, confinement period at home, fear of evil spirits, lack of understanding of service provision, unreliable health facilities, cost	35
Tefera et al., 2014, Ethiopia	Illness recognition, home care, and care-seeking for sick infants less than two months of age in Shebedino District, Sidama Zone, Ethiopia	Qualitative Focus group discussions following review of sick children registers	Examine illness recognition, home care, decision-making, and care-seeking for sick, young infants, particularly to understand low use of curative services	60 mothers of recently ill children (6 focus groups of 10 mothers from 6 kebeles) under 2 months of age in Shebedino District	Traditional beliefs of causes of illness affected where care was sought Care-seeking generally delayed Lack of knowledge of health services and belief that NDS resolve spontaneously Other care-seeking barriers included distance, husband’s decision, elder’s advice, fear of evil eye or injection, perceived cost	32
Thairu et al., 2022, Zambia	Care-seeking behavior for Newborns in Rural Zambia	Qualitative Focus group discussions	Explore mothers’ healthcare-seeking related to newborn illness and reasons for delay in care seeking	60 mothers and 77 grandmothers of children < 3y in Lufwanyama District	Awareness of newborn vulnerability and practised confinement period led to a preference for home care Perceived severity affected care-seeking Multiple care-seeking steps were common Distance, perceived lack of respectful treatment, costs of medication and fear of newborn dying in facilities hindered care-seeking	35
Vanosdoll et al., 2019, Uganda	A Novel Mobile Health Tool for Home-Based Identification of Neonatal Illness in Uganda: Formative Usability Study	Qualitative/usability study Semi-structured interviews with mothers, focus group discussions with volunteer Community Health Workers following simulated device use	Explore the usability and acceptability of the NeMo system (neonatal assessment tool)	32 women with neonates and 12 Community Health Workers in Iganga-Mayuge district	NeMo device was useable and acceptable Mothers trusted the device Cost of device or smartphones were perceived barriers	33

**Table 5.** Mixed-methods studies included.

Author, date, country	Title	Study design + method	Aim	Sample	Main findings	Quality score/36
Abate et al., 2022, Ethiopia	Knowledge and health seeking practice of mothers on neonatal danger signs and its associated factors at East Belesa Woreda, Northwest Ethiopia, 2020	Cross-sectional study with a qualitative component Structured questionnaire plus semi-structured interviews	Assess mothers’ knowledge about NDS and health-seeking practice	624 mothers with babies under 1 year of age, plus 11 mothers, 5 health Extension Workers, 3 priests and 2 shahs for interviews from East Belesa area	74% mothers had heard of NDS Higher educational level, antenatal care attendance, previous experience of NDS, good essential newborn care practices, and birth in a facility were associated with good knowledge of NDS	**34**
Assefa, 2014, Ethiopia	Assessment of knowledge and health care seeking behaviour about neonatal danger signs among mothers visiting immunization unit in selected Governmental Health Centers, Addis Ababa, Ethiopia	Cross-sectional study with qualitative component Structured questionnaire	Assess mothers’ knowledge and care seeking behaviour about NDS	373 mothers from 16 health centres in Addis Ababa attending immunization clinics	Higher education level of father and having recent experience of a sick neonate were associated with good NDS knowledge 65% sought health-care for a sick neonate Knowledge of NDS was associated with good care-seeking Delayed care-seeking mostly due to belief child would recover spontaneously	**31**
Bogale et al., 2017, Ethiopia	Why gone too soon? Examining social determinants of neonatal deaths in northwest Ethiopia using the three-delay model approach	Mixed methods Social autopsy of 39 neonatal deaths using HDS data of neonatal deaths that occurred in the previous 18 months	Investigate delays in care seeking associated with neonatal deaths	Data/family members related to 39 neonatal deaths in northwest Ethiopia	Median time from symptom recognition to seeking care was 1 day Delay in treatment-seeking was associated with 81% of deaths Reasons for delayed care-seeking: newborn not old enough to receive treatment, confinement at home, expected to resolve spontaneously, illness at night-time	**35**
Feyisso et al., 2016, Ethiopia	Assessment of knowledge of mother on danger signs of neonatal and postnatal illness and health seeking behaviour among pregnant and postpartum mother in Gedeo Zone, 2014/15	Cross-sectional study with qualitative component Structured questionnaire, plus in-depth interview and focus group discussions	Assess the knowledge of mothers on danger signs and health seeking behaviour	700 pregnant or postpartum mothers residing in Gedeo Zone (time since birth undefined)	32% knew 1+ NDS Fever was the most known NDS	**22**
Gathoni, 2014, Kenya	Mother’s knowledge, attitude and practice regarding neonatal illness and assessment of Neonates at Kenyatta National Hospital	Mixed-methods Structured questionnaire + focus group discussions	Assess knowledge, attitudes, and practice towards recognition of NDS among mothers	384 mothers in postnatal ward in Kenyatta National Hospital in Nairobi	Mothers were concerned when babies had fever, were not feeding well or looked sick Age, parity and education were associated with NDS knowledge Cultural beliefs affect parents’ perceived cause of illness and treatment Home care usually tried before hospital	**33**
Matin et al., 2020, Uganda	Feasibility of a Mobile Health Tool for Mothers to Identify Neonatal Illness in Rural Uganda: Acceptability Study	Mixed-methods Mothers trained to use the NeMO system and used the system at home for 1 week, use tracked by smartphone/observation Feedback gained from interviews	Determine if mothers in rural Uganda were willing and able to use the NeMo system and assess mothers’ responses to the device’s recommendations	20 mothers who had given birth in Iganda District Hospital	All mothers found the NeMo system easy to use and helped them know when to seek care Some mothers needed support from the study team to use the device effectively	**34**
Odundo et al., 2021, Kenya	Efficacy of a discharge checklist for neonates in reducing neonatal morbidity and mortality	Mixed methods Quasi-experimental pre-post intervention design, with focus group discussions, structured questionnaires and checklists	Determine the impact of using a standardised neonatal checklist	435 mothers of well neonates on postnatal ward in Kenyatta National Hospital, Nairobi (216 pre-, 219 post-intervention) plus 3 focus group discussions	Intervention significantly increased knowledge of fever as NDS Intervention significantly decreased knowledge of ‘other’ NDS symptoms Checklist was acceptable to nurses	**26**
Sibley et al., 2017, Ethiopia	Appropriateness and timeliness of care-seeking for complications of pregnancy and childbirth in rural Ethiopia: a case study of the Maternal and Newborn Health in Ethiopia Partnership	Mixed methods Secondary analysis of data from the 2010–2012 surveys and illness narratives	Present the Maternal and Newborn Health in Ethiopia Partnership Case study and explore differences in care seeking for maternal and neonatal illness	1027 and 1019 Ethiopian women of reproductive age who gave birth within one year before each survey and 29 mother-baby dyads who had experienced newborn illness	Care seeking was more common for mothers than neonates Multiple steps taken before attending a health facility Even when illness considered serious care was not always sought Barriers to care-seeking were time of day, weather, distance, transport, cost, mothers’ condition, baby’s condition, local beliefs requiring local healers, belief the baby would get better, fear of the evil eye, restricted movement during postnatal period, belief no medical treatment suitable Care-seeking more likely with referral from community health worker, close proximity to the facility, or when parents feared traditional medicine	**34**

### Quality appraisal

Although quality assessment is inherently subjective and relies on information provided by the authors, the use of a systematic tool by SF ensured a rigorous evaluation of all study elements. The tool developed by Hawker et al. [20], while less detailed than frameworks designed for single-study designs, proved effective in assessing quality across a variety of study types. Among the seventy studies, sixty-four were classified as high quality, five as medium quality, and one as low quality. No study was excluded based on quality assessment.

## Theme 1: poor knowledge of neonatal illness

The first theme presents aspects associated with parents’ lack of understanding of neonatal illness. Thirty-nine studies contributed to the development of the sub-themes, the majority were cross-sectional studies, conducted in Ethiopia, and focussed on mothers.

### Subtheme: parents find neonatal illness difficult to recognise

Although this literature review explored general knowledge about neonatal illness, rather than the symptoms of neonatal illness specifically, the World Health Organization definition of NDS was often adopted by studies. Parents were most aware of fever, followed by difficulty breastfeeding or breathing [22,23]. Recognition of local infections, convulsions, hypothermia, and jaundice was less common [24–26]. ‘Good’ knowledge of NDS lacked a consistent definition. Knowledge of at least three NDS varied from 0.7% [23] to 63.4% [27] but was affected by whether parents were prompted [28]. In Kenya, fathers (*n* = 82) and mothers (*n* = 348) demonstrated similar knowledge (50% versus 51% knew one NDS) but sample sizes and recruitment strategies differed between the men and women [15]. Qualitative studies reported that while parents understood newborn vulnerability to illness [29], they often found illness difficult to recognise [30,31]. Symptoms were often attributed to ‘local’ or spiritual problems [32].

### Subtheme: knowledge correlated with maternity and socio-economic factors

Health professionals were the primary source of information about NDS, followed by media, friends and relatives, and literature [25,33]. Specific NDS education was uncommon [22,28,34] but was positively associated with parents having ‘good’ knowledge of NDS [34,35]. Antenatal care, facility birth and postnatal care attendance were significantly associated with knowledge of NDS, even when there was no specific NDS education included [22,24]. Mothers with higher educational levels, employment outside the home, higher income, and urban residence tended to have better knowledge [28,36,37]. Previous experience with a sick neonate [38,39] and increased parity [40,41] were also associated with better knowledge. Qualitative studies supported these findings [31,42].

This theme has identified that while parents’ knowledge of specific NDS varies, they sometimes have inadequate knowledge and find neonatal illness difficult to recognise. Cultural beliefs about perceived causes of illness affected parents’ understanding. Routine health education during maternity or postnatal care often lacked inclusion of NDS, yet attending these was associated with better NDS knowledge. Additionally, better knowledge of NDS was associated with socio-economic factors such as education, occupation, and income.

## Theme 2: sub-optimal healthcare-seeking behaviour

The second theme explores parents’ care-seeking behaviour for neonatal illness. Twenty-five studies addressed elements of care-seeking, highlighting varied care-seeking incidence, cultural beliefs impeding care-seeking, delayed healthcare utilisation, and factors associated with care-seeking behaviour.

### Subtheme: delayed healthcare utilisation

‘Appropriate’ care-seeking varied and was poorly defined. Ethiopian illness narratives (*n* = 22 maternal, 29 neonatal), found that care-seeking for neonates occurred less often and later than for mothers [43]. In Kenya, half of the parents sought care promptly within an hour, while one-third waited over six hours [15]. In Ethiopia, some parents waited more than two days [33]. Delays were often attributed to waiting for neonatal improvement or seeking care only when the illness worsened [35]. Indeed, in Rwanda, over 40% of neonatal deaths (*n* = 1324) were associated with delays [44], and an Ethiopian study (*n* = 37) found that 81% of deaths were related to delays [45].

### Subtheme: complex interplay of cultural, social, and economic factors affected care-seeking

**Figure 2.**
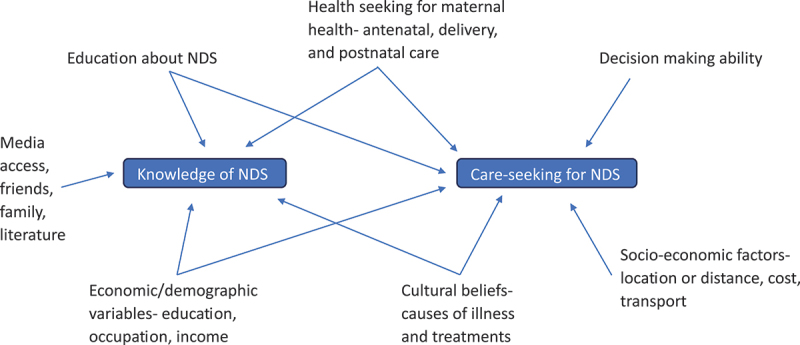
Factors associated with parents’ knowledge of NDS and care-seeking behaviour.

Maternity care attendance, education, income, and distance from health facilities were associated with care-seeking [46,47]. Knowledge of NDS and the ability to make autonomous decisions also played a significant role [33,46,48]. Conversely, barriers included cost, workload, transport, weather, and distance [43]. A lack of awareness about treatment options [24], adherence to traditional postpartum home confinement periods, and perceptions of non-serious illness contributed to delays [27,43,49]. Favouring traditional healers and home remedies [33,45], along with previous negative experiences with healthcare providers, reduced care-seeking [27,47]. In Ethiopia, approximately 70% of health-seeking behaviour could be predicted by knowledge of NDS (β = 0.41, *p* < 0.001), positive perceptions of healthcare providers (β = 0.08, *p* < 0.002), household-level women’s empowerment (β = 0.18, *p* < 0.001), and perceived treatment costs (β = 0.06, *p* < 0.002) [50]. Concerns about maternal empowerment led to studies exploring mothers’ decision-making abilities. However, these concerns were largely unfounded, as most parents made joint decisions, with some mothers deciding independently [27,46]. Fathers were typically involved because of their role in family financial provision, while it was uncommon for grandparents or other relatives to make healthcare decisions [33,48]. Factors associated with parents’ knowledge and care-seeking behaviour are summarised in Figure 2.

This theme identified that many parents do not demonstrate appropriate care-seeking for neonatal illness, sometimes delaying for long periods. Knowledge of neonatal illness affected whether parents sought healthcare, as well as cultural beliefs and norms, use of alternative treatments, perception of healthcare providers, or practical factors such as cost or transport.

## Theme 3: strategies to support parents’ understanding

This theme evaluates interventions that have been implemented in nine studies. Three studies evaluated interventions where community health workers sought to increase parents’ knowledge of NDS through postnatal assessment, home visits, and health education. Three studies evaluated the use of health promotion text messages, phone calls, or videos in increasing NDS knowledge, and a further study introduced and evaluated a standardised discharge checklist, to encourage health professionals to educate parents about NDS. Finally, two studies evaluated a neonatal monitoring system, where a smartphone app and wearable band were designed to support parents in identifying NDS.

### Subtheme: facilitation by community health workers

In Ethiopia, a quasi-experimental study of mothers who had given birth in the previous year (*n* = 705 baseline, 980 endline) found that postnatal checklists assisted community health workers’ neonatal assessments, and facility health worker training equipped them to educate mothers [51]. ‘Good’ knowledge (knowing three or more NDS) increased by 13.6% (*p* = 0.012). However, only 55% of the intervention group received the intervention, and not all individual danger signs showed significant improvement. Another quasi-experimental study (*n* = 2237 baseline, 1946 endline), in Uganda, found that community health worker visits, radio messages, and community meetings increased mothers’ NDS knowledge [52]. Knowledge of NDS increased from 37% to 65% in the control area, and from 43% to 91% in the intervention area, indicating a significant intervention contribution (Difference in Difference 20, *p* < 0.001). However, a large increase in knowledge occurred in both the intervention and control groups, suggesting other variables influenced knowledge during the study period. In the Democratic Republic of Congo, a quasi-experimental pilot study delivered home visits and education sessions to male partners (*n* = 600 comparison zones, 648 intervention zones) [53]. There was some crossover, with over one-third of men in the intervention zones not participating in the intervention and a small number (<5%) of men receiving the intervention from the comparison zones. Residence in the poorest households was significantly higher in the intervention zone (38%) compared with the comparison zone (27%). However, men in the intervention zones were more likely to know three or more NDS (Average Treatment Effect = 0.139, 95%CI 0.063–0.216, *p* < 0.001).

### Sub-theme: effective multi-media methods use

In Tanzania, a quasi-experimental study found an interactive mobile messaging service increased women’s NDS knowledge (intervention *n* = 150, control *n* = 300) [54]. The effect size was substantial (Cohen’s d = 0.85, *p* < 0.001). Similarly, in Kenya, a randomised controlled trial found mothers who received health education via text messages (*n* = 60), or phone calls (*n* = 60) demonstrated higher NDS knowledge scores (75.7% and 80.5%, respectively) than the usual care group (*n* = 60, 42.7%) (*p* = 0.016 text versus control, *p* = 0.008 phone versus control) [55]. Another randomised controlled trial in Kenya showed that mothers who watched an NDS video (*n* = 77) had significantly increased recognition of some NDS after compared to mothers who received an NDS handout (*n* = 76) [56]. After one week, mothers in the intervention group had greater knowledge of trouble breathing (OR 2.5 95% CI 1.25–5, *p* = 0.003) and red swollen eyes with drainage (OR 2.5 95% CI 1.4–5, *p* = 0.03). After four weeks the intervention group also had higher knowledge of fits (OR 2.5, 95% CI 1.1–5, *p* = 0.04), less energy (OR 2.5, 95% CI 1.25–5, *p* = 0.01), and skin pustules (OR 2.5 95% CI 1.1–5, *p* = 0.04).

Also in Nairobi, a mixed methods study evaluated the acceptability of a standardised neonatal discharge checklist which included education about NDS. In the quasi-experimental element, there was no significant difference between the proportion of mothers in the pre- (*n* = 216) and post-intervention (*n* = 219) groups who reported they would go to the hospital if they recognised NDS, as nearly all mothers indicated this (pre-intervention 91.3%, post-intervention 93%, *p* = 0.439). The study did find a statistically significant increase in knowledge of fever (67.4% to 79.8%, *p* = 0.05) but a decrease in knowledge of other NDS symptoms (32.6% to 20.2%,*p* = 0.05) in the post-intervention group. The ‘other’ symptoms were not defined, and the reasons for the decrease were not explained. Despite the study’s claims that the checklist was acceptable and potentially beneficial, the study quality was rated poorly in the data analysis and results sections.

### Subtheme: acceptable neonatal monitoring system

Two mixed method studies evaluated the usability and acceptability of a neonatal monitoring system (NeMo) in Uganda. NeMo combines a smartphone app with a wearable neonatal band to support parents in assessing NDS in the neonate. The NeMo band fastens around the neonate’s abdomen and measures respiratory rate and temperature. The app requires mothers to respond to audio and visual cues to assess other danger signs including difficulty breastfeeding, chest indrawing, convulsions, and lethargy. In one study, mothers (*n* = 32) used the device in a simulated setting and reported ease of use and efficacy in identifying NDS during in-depth interviews [57]. In another study, mothers used the device at home for one week (*n* = 20) [58]. While these mothers also found NeMo easy to use, some were not fully compliant and required ongoing support from the study team. The effects on neonatal outcomes have not yet been evaluated.

This theme found that interventions involving community health workers facilitating health education increased parents’ understanding of NDS. Providing mothers with health education text messages, phone calls, or videos was also effective, whereas using a standardised discharge checklist did not impact mothers’ intended care-seeking behaviour. A neonatal monitoring system combining a smartphone app and a monitoring band was acceptable and useable, so further testing must establish the effect on care-seeking and neonatal outcomes.

## Discussion

This integrative review explored the literature surrounding parents’ knowledge of neonatal illness, related care-seeking behaviour, and interventions to support improved parental understanding in the African Great Lakes countries. The findings highlighted a significant lack of knowledge among parents regarding neonatal illness. ‘Good’ knowledge was typically defined as recognising three out of at least nine NDS, indicating that even parents with ‘Good’ knowledge might be aware of less than a third of the symptoms. Fever in newborns was the greatest concern for parents, but there was limited discussion about parents holding incorrect knowledge. Addressing misconceptions is crucial when designing effective interventions, so future research must explore these. Factors associated with better NDS knowledge included attendance at maternity care, higher education levels, higher income, and urban residence. Mothers with prior experience caring for sick neonates also exhibited better knowledge. However, most studies included were cross-sectional, using analytic methods that cannot establish cause-and-effect relationships, warranting more sophisticated analyses in future studies. Also, questionnaire-based and qualitative studies conducted many months postpartum relied on parents’ recollections, which are prone to recall bias. While health workers were the primary source of information, most women received little or no education about NDS during antenatal, birth, and postnatal care interactions. Postnatal care is very limited in many sub-Saharan African settings [59]. Barriers to health education opportunities during other maternity contacts are not yet fully understood [60].

Parents’ lack of knowledge about NDS correlated with poor care-seeking behaviour. In contrast, mothers who attended maternity care, had higher education and income, or were from urban areas were more likely to seek care. These findings align with a multi-country study (*n* = 31 sub-Saharan African countries) examining mothers’ care-seeking for older infants, which found education, occupation, and wealth were associated factors, although urban residence was negatively associated [61]. While other studies have highlighted the vital role of grandmothers in newborn care [62], this review found that most mothers made decisions regarding the neonate alone or with the father. However, parents were less likely to seek care for the neonate than for the mother. The studies did not provide any explanation, although, in some African communities with high neonatal mortality, neonates are not considered full individuals [63].

Cultural beliefs affected decision-making, with traditional healers and home remedies often used first. Across sub-Saharan Africa, traditional medicine is also widely used for pregnancy-related symptoms, yet a systematic review of 20 studies conducted in 12 African countries found its use was associated with lower education, lower income, or residing far from health facilities [64]. By focussing on single care-seeking elements, existing studies often overlooked the complex steps families took before reaching health facilities. Delays in the decision to seek care, and in attending a health facility, corresponded with elements of the Three Delays Model [65]. This identifies critical delays that can prevent women from accessing timely and effective maternal healthcare: the delay in deciding to seek care, the delay in reaching a healthcare facility, and the delay in receiving adequate care at the facility. This model helps in understanding and addressing barriers to maternal health services; however, it has been criticised for its inability to capture the complexity of health outcomes [66] with other studies identifying additional contributing factors [67]. For example, perceived quality of care or previous disrespect in health facilities affects care-seeking decisions [68]. Therefore, promoting respectful maternal and newborn care is essential [69,70]. Most studies that examined women’s empowerment to make health decisions found that women were able to make decisions either alone or with their partner. This contrasts with other studies which have highlighted the negative impact of traditional gender roles and power relations on women’s ability to make decisions such as the use of family planning, finances, and maternal or neonatal health service utilisation [71,72].

Intervention bundles involving community health workers using postnatal checklists, or delivering home visits and education sessions, enhance parents’ knowledge of NDS. Community health workers are present in all the African Great Lakes countries, although their roles, training and responsibilities vary [73]. Their effectiveness has been demonstrated in other areas, such as increasing the reach, uptake and quality of HIV services [74]. While maternal and newborn health are typically within their scope, further evaluation is required [75], particularly as they are often poorly trained and remunerated, and can be overburdened with multiple community health responsibilities [76–78]. Male involvement in maternal and neonatal health has also been associated with improved knowledge of NDS. Other studies have highlighted broader benefits of male involvement, including improving antenatal care, skilled birth and postnatal care attendance, birth preparedness, and maternal nutrition [79]. The positive impact of male involvement may be linked to traditional gender roles and expectations in lower- and middle-income countries, which may impact women’s autonomy to make health decisions [72]. Additionally, using text messages, phone calls, and videos has effectively increased mothers’ NDS knowledge. However, the use of the videos was only compared with the provision of an information sheet about NDS, rather than with ‘usual care’, which would have provided clearer insights into their effectiveness. The feasibility and acceptability of a neonatal monitoring system combining a smartphone app and a wearable neonatal band to support mothers’ identification of NDS have been established. Further research is necessary to determine its effectiveness concerning subsequent outcomes, including mortality, care-seeking behaviours, and cost.

## Strengths and limitations

Using a structured framework, systematic search strategy, and quality assessment tool ensured rigour, but relevant studies may have been missed, for example, by limiting the search by date, language, and geography. The ability to include research from different paradigms was a strength, as findings from qualitative studies gave additional insight into quantitative findings. Although the search strategy only involved one researcher, ongoing discussions with the supervision team and using strict inclusion and exclusion criteria prevented cherry-picking and ensured consistency. Although many studies were included, the study objectives would have been compromised if the searches had been restricted further.

## Conclusion and recommendations

This integrative review synthesised research from the African Great Lakes countries on parental knowledge and care-seeking for neonatal illness, as well as interventions aimed at improving their understanding. Qualitative studies revealed that parents often have a limited understanding of the causes of neonatal illness and the rapid health deterioration that can occur in neonates. Therefore, educational efforts must go beyond simply listing symptoms and focus on providing a more comprehensive understanding of neonatal illness. Promoting antenatal care, skilled birth, and postnatal care attendance remains critical, as these factors are associated with better parental knowledge and timely care-seeking. However, few women received specific health education during maternity care. Further research should explore the current state of health promotion within maternity care and identify the barriers and facilitators to increasing its reach. Additionally, understanding the role of women’s empowerment within different cultural contexts is essential, as interventions may need to address family dynamics and decision-making processes.

While this review offers insights into reasons for delayed care-seeking, further research is required to examine how cultural practices, beliefs, costs, convenience, family pressure, or personal values influence parental decisions. It is also crucial to capture the multiple steps parents take before accessing healthcare services. Some parents expressed reluctance to visit health facilities due to past negative experiences or unfavourable perceptions of healthcare staff. However, it is unclear whether these were personal experiences or general community perceptions, warranting further investigation. Interventions to enhance parents’ understanding of NDS have been limited, though some success has been achieved using community health workers and multi-media methods. While these methods may be transferable to similar contexts, further evaluation is necessary to confirm their effectiveness.

## Supplementary Material

PRISMA_2020_checklist for IR GHA.docx
